# The Planktonic Core Microbiome and Core Functions in the Cattle Rumen by Next Generation Sequencing

**DOI:** 10.3389/fmicb.2018.02285

**Published:** 2018-09-24

**Authors:** Roland Wirth, Gyula Kádár, Balázs Kakuk, Gergely Maróti, Zoltán Bagi, Árpád Szilágyi, Gábor Rákhely, József Horváth, Kornél L. Kovács

**Affiliations:** ^1^Department of Biotechnology, University of Szeged, Szeged, Hungary; ^2^Helvecia Protein Ltd., Szeghalom, Hungary; ^3^Institute of Plant Biology, Biological Research Center, Hungarian Academy of Sciences, Szeged, Hungary; ^4^Institute of Biophysics, Biological Research Center, Hungarian Academy of Sciences, Szeged, Hungary; ^5^Faculty of Agriculture, University of Szeged, Hódmezövásárhely, Hungary; ^6^Department of Oral Biology and Experimental Dental Research, University of Szeged, Szeged, Hungary

**Keywords:** rumen, whole genome, whole transcriptome, core microbiome, core functions, metabolic pathways, functions, taxa

## Abstract

The cow rumen harbors a great variety of diverse microbes, which form a complex, organized community. Understanding the behavior of this multifarious network is crucial in improving ruminant nutrient use efficiency. The aim of this study was to expand our knowledge by examining 10 Holstein dairy cow rumen fluid fraction whole metagenome and transcriptome datasets. DNA and mRNA sequence data, generated by Ion Torrent, was subjected to quality control and filtering before analysis for core elements. The taxonomic core microbiome consisted of 48 genera belonging to *Bacteria* (47) and *Archaea* (1). The genus *Prevotella* predominated the planktonic core community. Core functional groups were identified using co-occurrence analysis and resulted in 587 genes, from which 62 could be assigned to metabolic functions. Although this was a minimal functional core, it revealed key enzymes participating in various metabolic processes. A diverse and rich collection of enzymes involved in carbohydrate metabolism and other functions were identified. Transcripts coding for enzymes active in methanogenesis made up 1% of the core functions. The genera associated with the core enzyme functions were also identified. Linking genera to functions showed that the main metabolic pathways are primarily provided by *Bacteria* and several genera may serve as a “back-up” team for the central functions. The key actors in most essential metabolic routes belong to the genus *Prevotella.* Confirming earlier studies, the genus *Methanobrevibacter* carries out the overwhelming majority of rumen methanogenesis and therefore methane emission mitigation seems conceivable via targeting the hydrogenotrophic methanogenesis.

## Introduction

Ruminants are one of the most successful groups of herbivorous mammals on the planet, having evolved the forestomach, the rumen, where feed is degraded before it enters the true stomach and the rest of the digestive system. The rumen provides an environment for a diverse consortium of anaerobic microbes. These microbes produce enzymes which are needed to break down complex molecules, primarily plant polysaccharides (Dai et al., 2015). The decomposition of cellulose-rich fibers involves intimate symbiotic relationships in the microbiota (Moran, 2005). The hydrolysis of lignocellulose often limits the kinetics and efficiency of metabolite production (Chapleur et al., 2014; Güllert et al., 2016). During rumination, sophisticated mechanical pretreatment of the lignocellulosic substrate and the delicate oxygen gradient along the route of feed play important roles in making the degradation of recalcitrant substances more efficient (Bayané and Guiot, 2011). The rumen microbiome is a well-studied, although not thoroughly understood microbial ecosystem, which is able to utilize the plant material in an organized food chain (Krause et al., 2003; Chapleur et al., 2014). In the first step, *Protozoa, Fungi*, and *Bacteria* carry out the hydrolysis of polymers, such as cellulose and other complex carbohydrates, proteins and lipids to low molecular weight compounds (Lynd et al., 2002; Jindou et al., 2008; Güllert et al., 2016). Subsequently, fermentative bacteria convert these metabolites to short chain fatty acids (SCFAs) like acetate, propionate and butyrate, CO_2_, H_2_, alcohols and other compounds. Methanogenic *Archaea* produce methane in the final step of the microbial food-chain (Le Van et al., 1998; Hegarty and Gerdes, 1999; Lopez et al., 1999; Janssen and Kirs, 2008; Hook et al., 2010). These communities co-evolve with their host according to their ability to convert some of the SCFAs, methylamines, CO_2_ and H_2_ to CH_4_, CO_2_ and water (Jami and Mizrahi, 2012; Meale et al., 2012; Poulsen et al., 2013; Jewell et al., 2015). The host benefits most if the process generates SCFAs (Weimer, 2015), which are absorbed through the rumen wall and used in the biosynthesis of sugars, lipids, and amino acids for the animal. Nonetheless, the thermodynamic and redox balances function properly only if a certain amount of chemical energy is released in the form of biogas, i.e., a mixture of CH_4_ and CO_2_ (Ungerfeld, 2014). The synthesis of acetate and butyrate involves the formation of reduced co-factors that require re-oxidization by methanogens (Ungerfeld, 2015). Conversely, propionate fermentation, which is the main precursor of glucose biosynthesis in ruminants, competes with methanogenesis for H_2_ (Janssen, 2010). The release of CH_4_ is an energetic loss for the animal and a major factor in global climate change (Martin et al., 2010; Opio et al., 2013).

Our knowledge of rumen microbiology has been accumulated by employing culture-based techniques. Studies on isolated cultures have provided a basic understanding of the biochemistry of the isolated strains (Bryant and Burkey, 1953; Hungate, 1960, 1966; Hobson, 1969). Unfortunately, most of the microbial strains in complex habitats cannot be cultivated in pure cultures; therefore, these approaches are of little help when the goal is the elucidation of the relationships between community members. The advent of nucleic acid-based molecular technologies opened up a new culture-independent perspective in microbial ecology. The majority of microbial identification studies rely on 16S ribosomal RNA (rRNA) gene sequencing. The 16S rRNA gene exists across bacterial and archaeal taxa and contains both highly variable and conserved regions (Woese and Fox, 1977; Skillman et al., 2006; Case et al., 2007; Wu et al., 2012), making it suitable for phylogenetic analyses (Case et al., 2007; Rajendhran and Gunasekaran, 2011). Nevertheless, 16S rDNA sequencing has some drawbacks as it suffers from biased choice of primers, PCR errors, and does not give functional information (Oulas et al., 2015). The meta-omic approaches give a much broader genomic and functional profile generated directly from environmental samples (Venter et al., 2001, 2004; Tyson et al., 2004). “Next generation sequencing” (NGS) has emerged, which employs various chemical reactions for the rapid determination of DNA sequences (MacLean et al., 2009; Metzker, 2010; Goodwin et al., 2016), produces huge datasets from relatively short sequence fragments and uses sophisticated bioinformatics to analyze the results (Huson et al., 2007, 2011; Raes et al., 2007). Initially, the most widely used NGS method in the identification of rumen communities and its functional mechanisms was based on 454-pyrosequencing (Mccann et al., 2014; Weimer, 2015; Güllert et al., 2016; Kala et al., 2017). Nowadays, other NGS approaches like Illumina^TM^, and Ion Torrent^TM^ have also been employed to investigate these communities (Singh et al., 2014; Mao et al., 2015; Pitta et al., 2016; Comtet-Marre et al., 2017).

At low resolution, the ruminal community appears quite stable (Russell and Rychlik, 2008). Studies have pointed out that diet is the major factor, which has more of an influence than animal species on the rumen microbiome (Jami and Mizrahi, 2012; Henderson et al., 2015; Kala et al., 2017). Nevertheless, there is still limited information available about the core rumen microbiome, i.e., microbes that are shared between individual animals, and about the common functional elements comprising the metabolic networks (Shi et al., 2014; Henderson et al., 2015; Li and Guan, 2017). The aim of this study is to contribute to our understanding of these aspects by examining 10 individual Holstein dairy cow whole metagenome and transcriptome datasets.

## Materials and Methods

### Animals

Ten multiparous cows in the second to fourth lactation period were sampled. The dairy cows were of the Holstein breed, selected from the herd maintained at the Pilot Farm, Faculty of Agriculture, University of Szeged, Hódmezövásárhely, Hungary. This study was carried out in accordance with the recommendations of Hungarian Law of Animal Protection and Charity XXVIII/1998 and Government Decree 40/2013 (II.14.) about animal protection. The protocol was approved by the Ethical Committee of the Faculty of Agriculture, University of Szeged. The best practice veterinary care has been followed and informed consent has been granted by the University of Szeged. The animals were fed with a mixed forage-concentrate diet, using a forage wagon, and water *ad libitum*. **Supplementary Table S1** shows the composition of the diet.

The stomach tube method was used to obtain rumen samples. The plastic collecting tube was 25 mm in diameter and was operated by manual aspiration of rumen fluid. The first 300 mL was discarded to avoid possible contamination with saliva. Sterile plastic containers were filled with 300 mL of rumen contents, flushed briefly with CO_2_ gas, snap-frozen in liquid nitrogen and transported to the laboratory within 1 h. The samples were stored at -20°C. After thawing, the material was filtered through a sterile mesh to remove suspended solids and used for further processing.

### DNA and RNA Isolation for Meta-Omic Studies

For total community DNA isolation, 2 mL of rumen liquid samples were used. DNA extractions were carried out using a slightly modified version of the Zymo Research Fecal DNA kit (D6010, Zymo Research, Irvine, CA, United States). The lysis mixture contained 100 μL CTAB (cetyltrimethylammonium bromide) to improve the efficiency (Wirth et al., 2015a,b). After lysis (bead beating), the Zymo Research kit protocol was followed.

For total RNA isolation, 1 mL of rumen liquid samples were taken. The RNA extractions were carried out with the Zymo Research Soil/Fecal RNA kit (R2040, Zymo Research, Irvine, CA, United States). After lysis (bead beating), the Zymo Research kit protocol was followed. The DNA contamination was removed by Thermo Scientific Rapidout^TM^ DNA removal kit (K2981, Thermo Fisher Scientific, Waltham, MA, United States).

The quantity of DNA and RNA was determined in a NanoDrop ND-1000 spectrophotometer (NanoDrop Technologies, Wilmington, DE, United States) and a Qubit 2.0 Fluorometer (Life Technologies, Carlsbad, CA, United States). DNA purity was tested by agarose gel electrophoresis and on an Agilent 2200 TapeStation instrument (Agilent Technologies, Santa Clara, CA, United States). The quality of the RNA preparation was checked by agarose gel electrophoresis (data not shown).

### Next Generation Sequencing and Data Handling

In sample preparation for total metagenome and metatranscriptome sequencing, the recommendations of the Ion Torrent PGM^TM^ sequencing platform were followed (Life Technologies, United States) using Ion Torrent PGM^TM^ 316 chips. Before metatranscriptome sequencing, rRNA was depleted from metaRNA by using the Gram+/Gram- depletion kit in 60:40 ratio (RiboMinus A15020 Life Technologies, United States).

The sequences which were produced by Ion Torrent PGM were denoised, normalized and sequencing artifacts were removed by MG-RAST software pack (DRISEE, Dinamic Trim) (Cox et al., 2010; Meyer et al., 2011; Keegan et al., 2012). The filtered data were downloaded and were further analyzed by Diamond applying default LCA (Lowest Common Ancestor) algorithm (Buchfink et al., 2014). Diamond was set as follows: Blast Mode: BlastX, Min Score: 50, Max Expected: 0,01, Top Percent: 10, Min Support: 1. The data were further analyzed by Megan6 (Huson et al., 2007, 2011, 2016). The NCBI Taxonomy database was used for taxonomic alignment, which is a standard nomenclature and classification repository for the International Sequence Database Collaboration (INSDC) comprising the GenBank, ENA (EMBL) and DDBJ databases (Federhen, 2015). For functional annotations, the InterPro database was applied. This database integrates several pieces of information about protein families, domains and functional sites (Finn et al., 2017). For carbohydrate active enzyme identification, the CAZy database was used^1^ (Lombard et al., 2014). Computing core microbiome and functions Megan6 co-occurrence plot function was applied with the following parameter sets: Threshold (%): 0.01, Min Prevalence (%): 0 Max Prevalence (%): 100, and Probability (%): 100. For functional statistic calculations, the R software was used^2^. The metatranscriptomic sequences were used to link functions to taxa by exporting the data from Megan and further analysis by the R software. The detailed sequence fragment parameters are summarized in **Supplementary Table S2**. The raw data were deposited in NCBI SRA database under the submission SRP148947.

## Results

### Metagenomic Profiles

**FIGURE 1:**
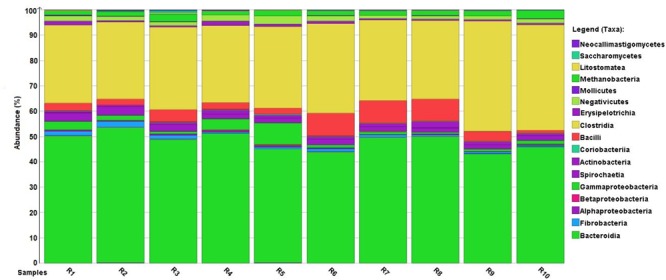
Normalized distribution of metagenome reads among *Bacteria* classes. Although the absolute number of reads (**Supplementary Table S2**) varied among individual rumen samples (R1–R10), the normalized values indicate a fairly consistent distribution of major taxa.

Sequencing genomic rumen DNA by Ion Torrent PGM produced an average of 305,884 reads with an average length of 168 bp. In average, 286,786 reads passed the quality control performed by the MG-RAST software package. These were subjected to quality control and were further analyzed by Diamond and Megan6. An average of 106,809 reads was obtained as known taxonomy units based on NCBI Taxonomy. Taking into account that 10 rumen samples were involved in the analysis, the overall read number exceeded 1 million (**Supplementary Table S2**). The most widespread phyla among *Prokaryota* are *Bacteroidetes, Firmicutes, Proteobacteria, Euryarchaeota*, and among *Eukaryota* are *Ciliophora, Ascomycota*, and *Neocallimastigomycota*. The taxonomic distribution at class level showed that the most abundant classes within *Bacteria* were *Bacteroidia* followed by *Clostridia, Bacilli, Spirochaetia* and *Gammaproteobacteria* (**Figure 1**). Within *Archaea*, the class *Methanobacteria* prevails. *Litostomatea, Saccharomycetes* and *Neocallimastigomycetes* are the most abundant classes among *Protozoa* and *Fungi*. Rarefaction analysis was performed at genus level. The curves reach its asymptotic nature, indicating that the sequencing depth at this scale is adequate (**Supplementary Figure S1**).

#### Core Microbiome of the Rumen

**FIGURE 2:**
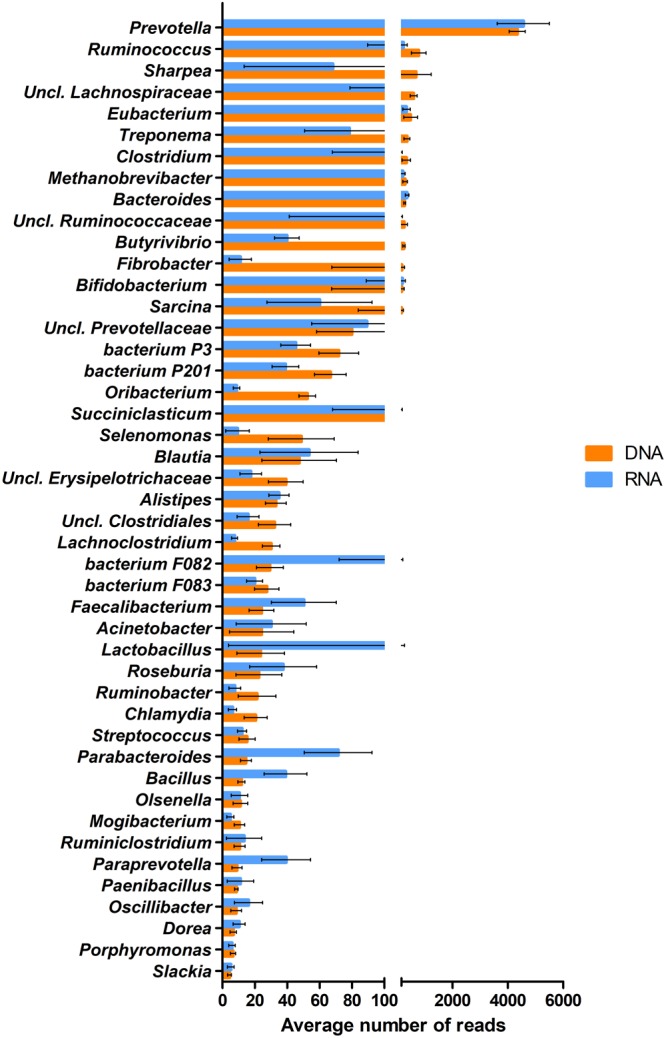
The abundance distribution of the 48 genera comprising the core microbiota calculated from the average DNA-based metagenome and RNA-based metatranscriptome datasets. Error bars indicate the variances among the individual 10 rumen samples.

Hundreds of microbial genera were identified after the double filtering approach, corroborating that the rumen microbiome is indeed a very diverse community. Co-occurrence analysis was used both on DNA and RNA data to select the planktonic core microbiome, i.e., the group of microbes found in all 10 rumen fluid samples, which were metabolically active. The core included 48 genera (**Figure 2**), which comprised about 18 and 23% of all genera, based on DNA and RNA sequences (**Supplementary Figure S2**).

The class *Bacteroidia* of the phylum *Bacteroidetes* contributed the highest number of core DNA sequences, although the species diversity of this group was relatively low. *Prevotella* prevailed over other *Bacteroidia* genera. The genus *Bacteroides* was among the top ten abundant genera in this class. The phylum *Bacteroidetes* contained unknown and uncultured species, like *bacterium P3, P201, F082, F083* or the uncultured class *Prevotellaceae* (Stevenson and Weimer, 2007) (**Figure 2**). *Alistipes, Parabacteroides, Paraprevotella*, and *Porphyromonas* were less frequent in the class *Bacteroidia* based on DNA data. Nevertheless, it should be noted that from these genera *Parabacteroides, Paraprevotella*, and from the uncultured and unclassified species *bacterium P3, P201* and *F082* showed high metabolic activity in RNA data.

The class *Clostridia* (phylum *Firmicutes*) produced fewer sequences in the core but was the most diverse higher taxon in the rumen fluid. Considerable amount of DNA sequence reads pointed at representatives of the genera *Ruminococcus, Eubacterium, Clostridium, Butyrivibrio, Oribacterium*, and *Sarcina*. Other genera such as *Blautia, Lachnoclostridium, Faecalibacterium, Roseburia, Mogibacterium, Ruminoclostridium, Oscillibacter*, and *Dorea*, were detected at a lower abundance. The representatives of genera *Butyrivibrio, Oribacterium*, and *Lachnoclostridium* had lower apparent metabolic activity than their DNA data suggested. Unclassified sequences were also found, which belonged in the families *Ruminococcaceae, Lachnospiraceae* and the phylum *Clostridiales*. It is noteworthy that more than 13 genera of the core 48 taxa belonged in the class *Clostridia* (**Figure 2**).

Also belonging in the phylum *Firmicutes*, the class *Erysipelotrichi* contributed to the planktonic core rumen microbiome with unclassified *Erysipelotrichaceae*, although this class was represented in relative small numbers. The genera *Lactobacillus, Streptococcus, Bacillus*, and *Paenibacillus*, in the class *Bacilli*, and the genus *Mycoplasma* (class *Mollicutes*) were barely detectable in the DNA based core microbiome. Few sequences of the genera *Succiniclasticum* and *Selenomonas*, cataloged in class *Negativicutes*, from which *Selenomonas* had a few related functions in the functional core compared to the DNA based hits (see Metatranscriptomic Profiles).

The class/phylum *Spirochaetes* was represented by the highly abundant genus *Treponema.*

The genus *Fibrobacter*, belonging in the phylum *Fibrobacteres*, class *Fibrobacteria*, and genus *Bifidobacterium* in the phylum/class *Actinobacteria* were also identified within the core genera. Surprisingly, significantly less RNA sequences mapped to the genus *Fibrobacter* than in the DNA data. Among the minor components of the functionally active core microbiome were other members of *Actinobacteria*, such as the genera *Olsenella*, and *Slackia*.

From the phylum *Proteobacteria*, class *Gammaproteobacteria*, the genera *Acinetobacter* and *Ruminobacter* were identified. The genus *Chlamydia* (class/phylum *Chlamydiae)* was observed in diminishing quantity in DNA reads, and it had even smaller number of RNA hits.

About 1% of all the identified sequences belonged in the phylum *Euryarchaeota*, class *Methanobacteria*. Only the genus *Methanobrevibacter* was verified from this taxon among the core microbes. It is noteworthy that, a comparable number of RNA and DNA sequences were mapped to the genus *Methanobrevibacter*.

The protozoa content of the rumen was about two orders of magnitude lower in cell number than the bacterial cell number (∼0.002%). The genus *Entodinium* (protozoan phylum *Ciliophora*, class *Litostomatea*) found its place in the DNA based planktonic core microbiome.

### Metatranscriptomic Profiles

#### Metabolic Pathways

Ion Torrent PGM RNAseq produced an average of 680,886 reads with an average length of 185 bp. In average, 661,310 reads passed the MG-RAST software quality control (**Supplementary Table S2**). From the mRNA sequences, in average 296,618 contained predicted protein coding regions with known functions, based on the InterPro database.

Most of the reads were related to metabolic or electron-proton exchange activity. Enzymes involved in biosynthetic, small molecule metabolism, carbohydrate metabolism and redox activities were among the most frequent. Housekeeping functions like translation, transport, RNA, DNA and amino acid metabolism were less persistent. The multifaceted picture was confirmed when putative protein sequences derived from the mRNA data set were projected onto KEGG metabolic pathways providing an integrated picture of global cell metabolism. The metabolic pathways showed that in the rumen fluid planktonic microbiome, the metabolism of carbohydrates and volatile fatty acid biosynthesis predominated (**Supplementary Figures S3–S6**).

#### CAZymes

**FIGURE 3:**
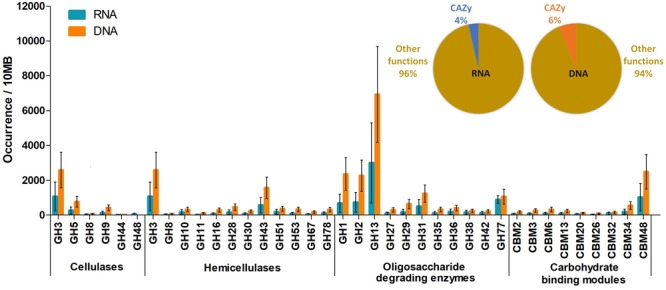
Glucoside hydrolase enzyme families identified in the DNA and RNA datasets, respectively. The inset indicates the proportion of GH sequences relative to the total read numbers.

A great number of cellulases, hemicellulases, and oligosaccharide-degrading enzymes were identified when the filtered reads were probed against the CAZy database. The share of putative CAZymes was 4% in the total RNA database (**Figure 3**). It is noteworthy that DNA-based metagenome data gave similar results, with 6% of DNA reads coding for carbohydrate-degrading genes (**Figure 3**). Moreover, the order of identified enzyme families showed similar tendency. Differences habitually appeared in the relative numbers of corresponding genes; the DNA sequences gave more matches compared to the RNA data. This may be explained by the difference in the number of coded and expressed genes. It is conceivable that not all of the genes in the genome are expressed at all times. Nevertheless, similar trends in the two sets of data clearly indicate that metagenomic DNA data, obtained by NGS, can provide useful information on the main metabolic functions. Among the CAZymes, five glycoside hydrolase (GH) families were prevalent among cellulases in our rumen samples, i.e., GH3, GH5, GH8, GH9, GH44, and GH48. The GH3, GH5, and GH9 families were the most copious among cellulases (**Figure 3**). The GH3, G5 and GH8 families are widely distributed in bacteria and fungi and have a variety of hydrolyzing activities. The GH9 and GH44 families mainly contain endoglucanases, while GH48 is a reducing end-acting cellobiohydrolase. Eight GH families (GH3, GH8, GH10, GH11, GH16, GH28, GH30, GH43, GH51, GH53, GH67, and GH78) are primarily hemicellulose degraders, and have both endo-acting and debranching enzymes. The identified frequent oligosaccharide-degrading GH families are as follows: GH1, GH2, GH13, GH27, GH29, GH31, GH35, GH36, GH38, GH42, and GH77. GH1, GH2, GH13, and GH77. Carbohydrate-binding modules (CBMs) are specialized domains in plant cell wall polysaccharide-degrading enzymes. They promote the intimate attachment of catalytic domains to the substrates and enhance the activity of the enzymes^3^. Nine CBM families, CBM2, CBM3, CBM6, CBM13, CBM20, CBM26, CBM32, CBM34, and CBM48, were identified in both the DNA and mRNA databases. CBM2, CBM3, and CBM6 are specific for cellulose, CBM20, CBM26, and CBM34 have a preference for starch and CBM13, CBM32, and CBM48 families have oligo- and monosaccharide-binding functions. **Supplementary Table S3** contains the entire list of the identified CAZymes.

#### Core Metabolic Functions

**FIGURE 4:**
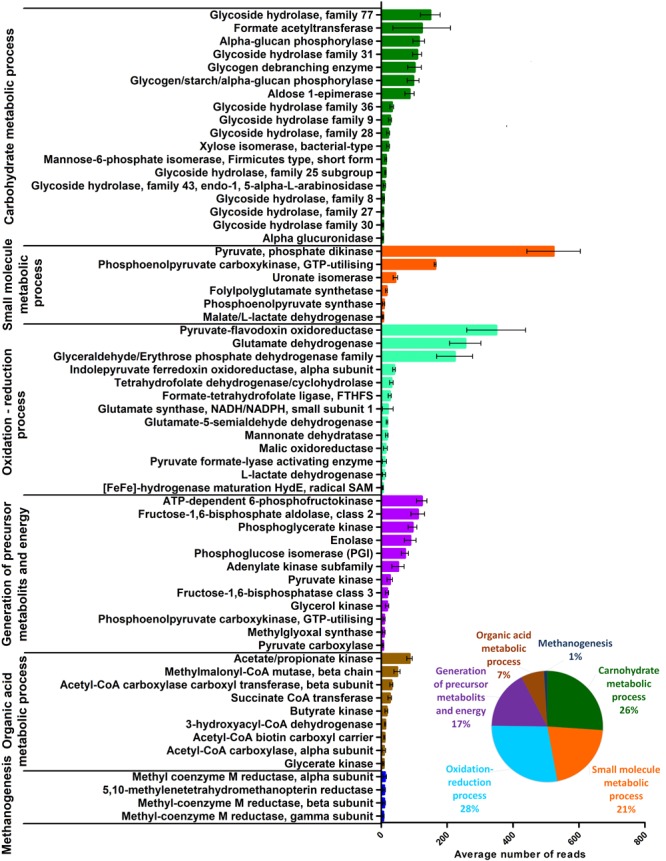
The distribution of the core microbial functions calculated from the average RNA-based metatranscriptome dataset. Error bars indicate the variances among the individual 10 rumen samples. The inset indicates the relative partition of the functional groups within the metatranscriptome.

Functional analysis of whole transcriptome data pinpointed thousands of active gene transcripts, confirming the metagenomic data from a distinct view, i.e., the rumen microbiome is a highly active metabolic system that is cumulatively equipped with copious numbers of biochemical functions. Co-occurrence analysis was performed to filter out the most common functions present in our samples. Filtering of the mRNA sequence database yielded at least 587 genes, which may form the core functions indicated by their interactions and presence in all data sets (**Supplementary Figure S7** and **Supplementary Table S4**). According to the InterPro database, 62 genes from this pool apparently code for enzymes involved in metabolic pathways (**Figure 4**). This is a small number for the enzymes to comply with the requirements of basic biochemical life-sustaining fermentative pathways, which is likely due to the stringent filtering conditions used in the co-occurrence analysis. The remaining transcripts are housekeeping genes required for the maintenance of basic cellular functions. The core metabolic processes provided satisfactory coverage of the entire microbiological food chain, from carbohydrate metabolism to methanogenesis. From the 62 core metabolic function coding genes, 18 belonged to carbohydrate processing, which comprised 26% of all core transcripts. Most of them encoded glycoside hydrolase families.

Among small molecule metabolic processes, pyruvate phosphate dikinase (PPDK) prevailed, which is a central glycolytic enzyme, together with phosphoenolpyruvate (PEP) synthase (PPsTK) (Berman and Chon, 1970; Imanaka et al., 2006). The second most frequently identified sequence indicated ATP-utilizing phosphoenolpyruvate carboxykinase (PEPCK), which participates in gluconeogenesis in co-operation with malate L-lactate dehydrogenase (Madern, 2002). Uronate isomerase is an enzyme involved in the intramolecular rearrangement of glucose (Williams et al., 2006). Folylpolyglutamate synthase might be implicated in the biosynthesis of the important methanogen intermediary tetrahydrofolate (Bognar et al., 1985). Additional enzymes found in the core functions complementing the tetrahydrofolate pathway include tetrahydrofolate dehydrogenase/cyclohydrolase (Pomper et al., 1999) and formate-tetrahydrofolate ligase (FTHFS) (Chistoserdova et al., 1998) (**Figure 4** and **Supplementary Figure S4**).

A diverse group of redox enzymes such as pyruvate-flavodoxin oxidoreductase (PFOR), glyceraldehyde/erythrose phosphate dehydrogenase family, the alpha subunit of indolepyruvate ferredoxin oxidoreductase, malic oxidoreductase, and L-lactate dehydrogenase indicated highly vigorous redox processes, which made up 28% of all core transcripts. Glutamate dehydrogenase, glutamate synthase and glutamate-5-semialdehyde dehydrogenase within this group signaled active ammonia assimilation (Yuan et al., 2009) by the rumen community. HydE, responsible for the maturation of [FeFe], hydrogenase suggests the presence of hydrogen metabolism in each rumen sample (**Figure 4** and **Supplementary Figure S6**).

A distinct group of the represented core functions take part in the synthesis of precursor metabolites and energy supply including ATP dependent 6-phosphofructokinase, fructose-1,6-bisphosphate aldolase, phosphoglycerate kinase, enolase, phosphoglucose isomerase, adenylate kinase and pyruvate kinase were found in high transcript numbers. Numerous additional enzymes were identified in the core functional group, which are also implicated in various biosynthetic pathways (**Figure 4**).

Overall, 7% of the total transcripts represented enzymes playing a part in organic acid metabolic processes, i.e., acetate/propionate kinase, methylmalonyl-CoA, acetyl-CoA carboxylase carboxyl transferase, succinate CoA transferase and butyrate kinase (**Figure 4** and **Supplementary Figure S4**).

Although they represent only 1% of the total core transcripts, Archaea enzymes performing methanogenesis play important functions in maintaining the proper redox balance in the rumen. Methyl-coenzyme M reductase alpha, beta, and gamma subunits and 5,10-methylenetetrahydromethanopterin reductase were detected in all of the rumen samples investigated (**Figure 4** and **Supplementary Figure S6**).

### Linking Core Functions to Taxa

In most metagenomic studies, the composition of the microbial community is determined and changes in relative abundances are recorded as the results of changes in various environmental conditions or feeding regimes. More recently, important experiments have complemented this information aiming at functional aspects by metatranscriptome, proteome and metabolome investigations concentrating on the “who does what?” question. The metabolic functions are grouped according to the InterPro classification^4^.

**FIGURE 5:**
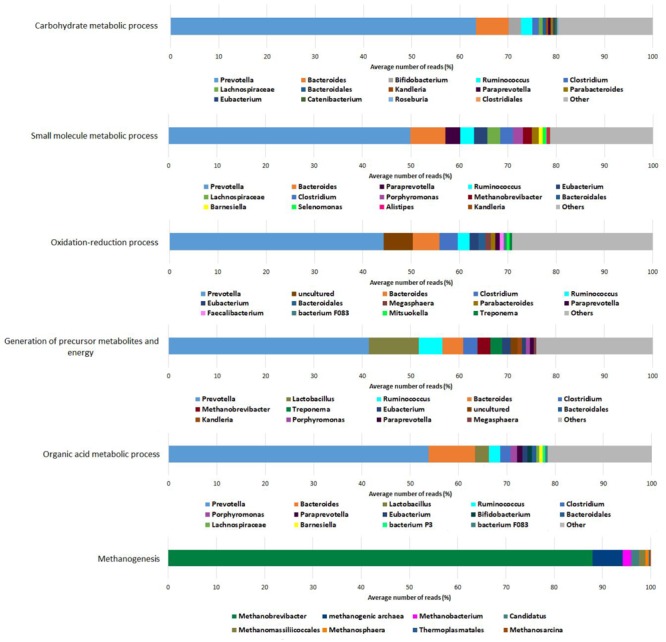
Coupling functional groups to taxonomic genera determined from the metatranscriptomic dataset.

In all of the functional groups associated with *Bacteria*, the genus *Prevotella* (phylum *Bacteroidetes*) prevailed (**Figure 5**), due to its vast predominance in the rumen planktonic microbiota and the versatile metabolic pathways possessed by this genus. Accordingly, the share of *Prevotella* in carbohydrate (∼63%), small molecule (∼50%), organic acid (∼53%) metabolisms, oxidation-reduction processes (∼44%), and generation of precursor metabolites (∼42%) was high, indicating that the majority of the metabolic “work” taking place in the rumen fluid is carried out by representatives of this taxon (**Figure 5**). Seven additional genera complemented the picture participating in the various metabolic pathways with varying activity. These were as follows: *Bacteroides, Ruminococcus, Bifidobacterium, Clostridium, Lactobacillus, Paraprevotella* and *Eubacterium*. It is interesting to note that the diversity ranking at the genus level is different from the abundance distribution based on the metagenomic data (**Figure 2**). The divergences between DNA and RNA based taxonomy data suggest that abundance and functional importance are not necessarily coupled.

The genus *Bacteroides* extensively contributed to carbohydrate, small molecule and organic acid metabolisms and played a significant role in other Bacteria-linked metabolic processes as well. The genus *Ruminococcus* was primarily active in the generation of precursor metabolites and energy although these species also participated in other bacterial tasks. *Clostridium* was more active in handling oxidation-reduction processes than *Ruminococcus*, and lagged behind *Ruminococcus* in carbohydrate metabolic process performance. In spite of their low relative abundance in the community, the genus *Lactobacillus* displayed remarkable contribution to tasks related to the generation of precursor metabolites and energy. *Bifidobacterium* showed considerable involvement in carbohydrate metabolism-related pathways and a noticeable one in organic acid metabolism.

*Paraprevotella* and *Eubacterium* were detected in the carbohydrate and small molecule metabolic processes. In the functional group “oxidation- reduction processes,” an uncultured bacterium was observed as the second dominant genus.

The genera *Kandleria, Parabacteroides, Porphyromonas, Selenomonas, Alistipes, Catenibacterium, Barnesiella*, and *Mitsuokella* were found less frequently among the various metabolic functions, perhaps due to their low abundance in the planktonic rumen microbiota. *Lachnospiraceae* and *Treponema* were the apparent exceptions as they were present at a relatively high number in the core rumen microbiome and proportionally low related activity.

In the methanogenesis-related core functions, the picture is completely different from the other functional groups, involving only archaeal taxa. In this function, the genus *Methanobrevibacter* predominated (∼88%). An uncultured methanogenic archaeon was also detected as the second most active in methanogenesis (∼6%). The contributions of the genera *Methanobacterium* (∼2%), an incompletely described archaeon genus *Candidatus* (∼2%), *Methanomassiliicoccales* (∼2%), *Methanosphaera* (∼1%), *Methanosarcina* (∼0.5%), and *Thermoplasmatales* (∼0.5%) were negligible.

## Discussion

### The Core Microbiome

The following discussion is restricted to genera present in the core microbiome and have functions associated to them (**Supplementary Table S4**). This information will be related to the results of previous rumen studies. In this study, our fundamental assumption has been that the taxa occurring together in the various rumen microbiota have shared functional connections and are therefore in some sort of metabolic relationship. This may not be the case in every individual interacting partner pair, but it is a reasonable postulation to find interrelationships between taxonomic and functional analyses in the core community.

Within the domain *Bacteria*, the majority of the identified sequences were annotated to the genus *Prevotella* (class *Bacteroidia*) (**Figure 2** and **Supplementary Figure S2**). Previous studies, using qPCR and/or 16S rDNA sequencing, also identified this genus as the most abundant in the rumen bacterial population (Stevenson and Weimer, 2007; Jami and Mizrahi, 2012). Further confirmation came from the analysis of the Sus-like (Starch utilization system) polysaccharide utilization loci (Naas et al., 2014; Rosewarne et al., 2014; Güllert et al., 2016). Changing dietary conditions did not alter *Prevotella* abundance (Stevenson and Weimer, 2007; Bekele et al., 2010; Purushe et al., 2010; Pitta et al., 2014; Lyons et al., 2017), although an age-related shift from *Prevotella* to *Succinivibrio* was noted recently (Liu et al., 2017). This is likely due to their ability to serve the wellbeing of their host in various ways, exploiting their high degree of genetic diversity (Avgustin et al., 1994; Ramšak et al., 2000) and remarkable metabolic versatility (Bryant et al., 1957; Wen et al., 1996; Matsui et al., 2000). In addition, *Prevotella* is a highly diverse taxon comprising various functional niches in different systems (Purushe et al., 2010).

The second most widespread group in the class *Bacteroidia* was the genus *Bacteroides*. This genus, like *Prevotella*, can utilize polysaccharides as an energy source. Apparently, some rumen *Bacteroides* can also decompose cellulose (Naas et al., 2014). Smaller genera in the class *Bacteroidia* include *Parabacteroides, Paraprevotella* and *Porphyromonas*, in addition to the *uncultured Prevotellaceae* family. These are usually detected in cow manure and their relative abundances change during the transition from developing and mature rumen (Dowd et al., 2008; Wu et al., 2012).

The classes *Bacteroidia* (phylum *Bacteroidetes*) and *Clostridia* (phylum *Firmicutes*) together represented ∼47-30% of the rumen core microbiome. In a recent study, the ratio of the two taxa in the whole rumen community was close to 1:1 (Güllert et al., 2016). Interestingly, in the biogas producing anaerobic microbial community metabolizing similar lignocellulosic substrates, *Clostridia* surpass greatly *Bacteroidia* (Güllert et al., 2016; Bozan et al., 2017). Apparently, the two classes differ in their colonization strategies. Only a few genera of the class *Bacteriodia* were present in the rumen, although in large relative abundances, whereas a remarkable diversity of genera characterized the class *Clostridia*.

Among *Clostridia*, the thoroughly studied genus *Ruminococcus* was frequently found. Their interactions with other species may make this genus more important than their abundance implies (Fondevila and Dehority, 1996; McSweeney et al., 1999; Koike and Kobayashi, 2009; Christopherson et al., 2014; Dai et al., 2015). Members of the family *unclassified Lachnospiraceae* and the genera *Eubacterium* and *Blautia* are among the taxa in the core of the cow rumen and present also in the kangaroo forestomach (Henderson et al., 2010; Godwin et al., 2014). These bacteria acquired the Wood-Ljungdahl pathway, which is alternatively called the reductive acetyl-CoA or the reductive acetogenesis pathway (Hattori, 2008; Ragsdale and Pierce, 2009; Xu et al., 2009; Gagen et al., 2015; Kelly et al., 2016) (**Supplementary Figure S4**).

The genus *Clostridium* possess high cellulolytic activity and actively produces H_2_ (Lin et al., 2007). The partial pressure of H_2_ determines the rate of methanogenesis and the assortment of short chain fatty acids generated in the rumen (Hegarty and Gerdes, 1999; Janssen, 2010). Methanogens outcompete the microbes involved in reductive acetogenesis because acetogens are less efficient at obtaining energy from the oxidation of H_2_ (Le Van et al., 1998; Siriwongrungson et al., 2007; Ungerfeld, 2015). The genus *Butyrivibrio* is present in a large selection of ruminants and has been characterized as an oligosaccharide-degrading bacterial taxon (Dai et al., 2015; Henderson et al., 2015).

The genus *Oribacterium* was observed as a particle associated bacterium in the rumen and the genus *Mogibacterium* was identified as a predominant member of the cattle gastrointestinal tract, although little is known about their functions (Mao et al., 2015; Schären et al., 2017). The genera *Faecalibacterium, Roseburia, Ruminiclostridium, Oscillibacter*, and *Dorea* were represented in low abundance. These diverse genera are active in cellulose and hemicellulose decomposition and ferment various sugars to short chain fatty acids (Duncan et al., 2006; Flint et al., 2008; Koike and Kobayashi, 2009; Thoetkiattikul et al., 2013; Ravachol et al., 2015).

The genera *Sharpea, Lactobacillus, Streptococcus, Bacillus*, and *Paenibacillus* represented the core members of the class *Bacilli* (phylum *Firmicutes*). Perhaps not surprisingly, the lactic acid bacteria *Streptococcus* and *Lactobacillus* tolerated aciduric challenge well in the cow rumen (Petri et al., 2013). Representatives of the genera *Bacillus* and *Lactobacillus* produce organic acids, some of which may endow probiotic effects (Qiao et al., 2010; Seo et al., 2013; Ushakova et al., 2013). The genus *Paenibacillus* has been noted for its hemicellulose-degrading activity (Kala et al., 2017), while the genus *Sharpea* tends to form and metabolize lactate an thereby increased its relative abundance in low methane yield sheep rumen (Kamke et al., 2016).

Within the core rumen planktonic microbiome, the genera *Succiniclasticum* and *Selenomonas*, were also present, belonging to the class *Negativicutes* (phylum *Firmicutes*), although in low abundance. The genus *Selenomonas* is lactate-utilizing bacteria (Hackmann and Firkins, 2015). *Succiniclasticum* members apparently differed in quantity between hay diet- and high grain diet-fed goats and have been described as starch degraders (Kim et al., 2011; Huo et al., 2014).

Three identified taxons in the core microbiome belonged in the class *Actinobacteria* (phylum *Actinobacteria*), these were the genera *Bifidobacterium, Olsensella*, and *Slackia*. Certain members of these genera are considered probiotic in both ruminants and humans as they metabolize oligosaccharides and release lactic acid, which helps to control the normal microflora (Picard et al., 2005; Nagai et al., 2010; Kamke et al., 2016).

The genus *Treponema* belong to the class/phyla *Spirochaeta*. Microbes in this genus are important fatty acid producers in the rumen (Yamada and Yukphan, 2008; Henderson et al., 2010; Li et al., 2012). The genus *Fibrobacter*, member of the class *Fibrobacteria* (phylum *Fibrobacteres*), contains well-known cellulose degraders (Dai et al., 2015). Our transcriptomic data indicate that the activity of this genus is lower than suggested by the DNA based hits. Similar phenomenon could be observed in case of the genera *Sharpea, Treponema, Butyrivibrio, bacterium P3, bacterium P201* and *Lachnoclostridium*. This may imply that, not always the predominant genera play essential role and some microbes present in small numbers may have significant activity in the microbial ecosystems.

In the rumen core microbiome the single genus *Methanobrevibacter* was found as the most prominent *Archaea* taxon. They are hydrogenotrophic methanogens and reduce CO_2_ to CH_4_ when H_2_ is present as an electron donor (Deppenmeier et al., 1996). The high rate of short chain fatty acid passage through the rumen wall via active uptake may explain the predominance of hydrogenotrophic *Archaea*, as the short chain fatty acid (SCFA) uptake is faster than the growth rate of acetate-utilizing methanogens (Dijkstra, 1994).

The total *Protozoa* and *Fungi* cell number in the rumen is two orders of magnitude lower than that of the *Bacteria*. In our data among *Protozoa*, the single genus *Entodinium* (class *Litostomateae*) positioned itself in the DNA based core microbiome, which reflects the uneven distribution of *Protozoa* in the individual rumen microbiota. The genus *Entodinium* is very efficient in starch fermentation and is a frequently detected protozoan in the rumen. (Jouany and Ushida, 1999; Zhang et al., 2017). Surprisingly, none of the *Fungi* were present in the DNA and RNA based core microbiome, suggesting that the fungal community is extremely varied across the individual rumen communities.

### Core Functions and Their Associated Microbes

**FIGURE 6:**
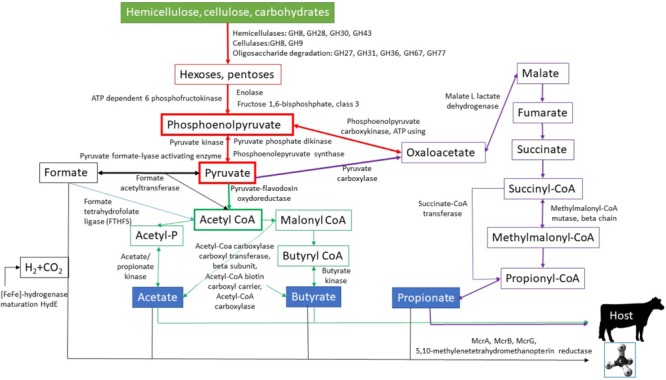
Reconstituted metabolic pathways involved in the decomposition of complex carbohydrates in the rumen. The enzyme names next to the arrows indicate enzymes identified in this work and their position in the metabolic network. For details see Section “Core Functions and Their Associated Microbes.”

Numerous studies recognized that the functional and phylogenetic distribution of microbes in the rumen comprise an integrated system; therefore, both aspects should be investigated together to better understand the complex metabolic processes taking place in this ecosystem (Deusch et al., 2015; Bielak et al., 2016; Mayorga et al., 2016; Shabat et al., 2016; Wang et al., 2016; Shen et al., 2017). In the present study, core metabolic functions and their accompanying microbes were determined from double filtered metatranscriptomic data by pairing the functional groups with the most probable taxonomic units. This resulted in a comprehensive coverage of the microbial food chains from complex carbohydrates to end products, i.e., SCFA and CH_4_, to be utilized or released by the host animal. In the following section, we therefore attempt to reconstruct a metabolic map of the physiological events taking place in the cattle rumen (**Figure 6**) and discuss the identified microbes associated with these biochemical reactions, extending the findings of similar previous studies (Stevens and Hume, 1998; Russell and Rychlik, 2008; Ungerfeld, 2014, 2015; Jiang et al., 2016; Deusch et al., 2017).

A great diversity of carbohydrate-active enzymes (CAZy) take part in the breakdown of the lignocellulosic feed components (Ferrer et al., 2012; Pope et al., 2012; Dai et al., 2015; Comtet-Marre et al., 2017; Kala et al., 2017; Svartström et al., 2017; Stewart et al., 2018). Our data corroborated these observations and numerous hemicellulose-, cellulose- and oligosaccharide-degrading enzymes, collectively called glycosyl hydrolases (GH), were detected in the first steps of the core energy providing metabolic pathways (**Figure 6**). GH8, GH28, GH30 and GH43 represent the hemicellulose degrading enzymes with polygalacturonase, xylanase and xylosidase activities. The genera *Prevotella* and *Bacteroides* predominate among microbes expressing these GH families (**Supplementary Table S3**). In the case of GH30, the genus *Prevotella* prevailed (∼30%), but other members of the class *Bacteroidia* also exhibited significant GH30 activity. The core cellulases might be GH8 and GH9. It is noteworthy that the GH9 family has been recognized before in numerous rumen ecosystems, indicating an important and general role in cellulose degradation (Brulc et al., 2009; Hess, 2011; Wang et al., 2013; Dai et al., 2015; Shinkai et al., 2016; Comtet-Marre et al., 2017). GH9 could be assigned primarily to the genus *Prevotella* (∼30%), although the genera *Bacteroides* (∼16%), *Ruminococcus* (∼12%), *Clostridium* (∼2%) and *Fibrobacter* (∼0.1%) expressed transcripts belonging in this enzyme family. Apparently, the oligosaccharide-degrading enzymes had the most diverse range of catalysts for carbohydrate breakdown. GH77 has amylomaltase and 4-α-glucanotransferase (EC 2.4.1.25) activity, which are mainly provided by the genera *Prevotella* (∼65%), *Ruminococcus* (∼3%) and *Clostridium* (∼3%). This GH family was found in the buffalo rumen among the highly active oligo-GH families (Patel et al., 2014). *Prevotella* and *Bacteroides* were the predominant genera harboring the enzyme families GH31 (*Prevotella*: ∼70%, *Bacteroides*: ∼11%), GH36 (*Prevotella*: ∼48%, *Bacteroides*: ∼8%), and GH27 (*Prevotella*: ∼50%, *Bacteroides*: ∼14%). Alpha-glucuronidases play an essential role in the complete hydrolysis of hemicellulose and were studied in *Bacteroides vulgaris* and *Dysgonomonas mossii* (Lee et al., 2012). The enzyme family GH67 was linked to the genera *Chryseobacterium* (∼29%), *Dysgonomonas* (∼10%) and *Paludibacter* (∼8%).

Taking the transcriptomic and taxonomic data together, the genera *Prevotella, Bacteroides* and *Ruminococcus* seem to predominate in the deconstruction of the lignocellulose rich feed. Previous reports highlighted that, in the rumen microbiota, the phyla *Firmicutes* and *Bacteroidetes* are almost equally responsible for cellulose degradation, while in hemicellulose decomposition the members of *Bacteroidetes* predominated (Comtet-Marre et al., 2017). Our data showed that *Prevotella* was primarily responsible for carbohydrate metabolism (**Supplementary Table S3**). *Prevotella* provided fewer transcripts of the cellulose GH9 family (∼30%) than those of the GH77 (∼65%), GH31 (∼70%), GH28 (∼57%), and GH43 (∼90%) enzymes, which are associated with oligosaccharide-degradation and hemicellulases.

Monomeric hexoses and pentoses are fermented to phosphoenolpyruvate in the subsequent metabolic steps (red boxes in **Figure 6**). Phosphoenolpyruvate is an important intermediary of glycolysis and gluconeogenesis (Parmar et al., 2015). We identified three genes in the core metabolic functions participating in this transformation, i.e., ATP-dependent 6 phosphofructokinase (IPR022953), enolase (IPR000941) and fructose-1,6-bisphosphate class 3 (IPR009164). ATP-dependent 6 phosphofructokinase catalyzed the transfer of a phosphoryl group from ATP; this is an important reaction in a wide variety of biological processes (Hellinga and Evans, 1987). This enzyme phosphorylates fructose-6-phosphate to fructose-1,6-bisphosphate; the reaction is a key regulatory step in the glycolytic pathway (Raben et al., 1995; Wegener and Krause, 2002). Our data suggested several taxa as the source of this enzyme activity, including the genera *Prevotella* (∼59%), *Bacteroides* (∼6%), *Ruminococcus* (∼4%) and *Kandleria* (∼4%). Enolase (2-phospho-D-glycerate hydrolase) is a homodimer enzyme that catalyzes the reversible dehydration of 2-phospho-D-glycerate to phosphoenolpyruvate in the presence of Mg^2+^ (Lebioda and Stec, 1991). The genera expressing this gene were *Prevotella* (∼39%), *Methanobrevibacter* (∼6%) and *Lactobacillus* (∼5%). Fructose 1,6-bisphosphatase converts fructose 1,6-bisphosphate to fructose 6-phosphate (**Figure 6**). This is a key reaction in gluconeogenesis (Marcus et al., 1986). The genera *Prevotella* (∼55%), *Bacteroides* (∼14%) and *Eubacterium* (∼5%) are the main suppliers of this activity. Phosphoenolpyruvate is converted to pyruvate in glycolysis or to oxaloacetate in the pathway leading to gluconeogenesis. In the reversible reaction, the pyruvate, phosphate dikinase (IPR010121), pyruvate kinase (IPR001697) and phosphoenolpyruvate synthase (IPR006319) participated (Berman and Chon, 1970; Imanaka et al., 2006), and were among the core metabolic functions. The highest number of transcripts found for pyruvate, phosphate dikinase and the related genera are principally *Prevotella* (∼68%), *Eubacterium* (∼3%) and *Ruminococcus* (∼3%). This enzyme catalyzes the reversible conversion of ATP and pyruvate to AMP and phosphoenolpyruvate (Evans and Wood, 1968).

Pyruvate kinase was found in the genera *Lactobacillus* (∼15%), *Prevotella* (∼15%), *Ruminococcus* (∼8%), *Kandleria* (∼8%), and *Eubacterium* (∼3%). This enzyme plays a role in the final step of glycolysis, i.e., the conversion of phosphoenolpyruvate to pyruvate (Muirhead, 1990). Phosphoenolpyruvate synthase was expressed predominantly by the genera *Prevotella* (∼32%), *Phasolarctobacterium* (∼26%) and *Acidaminococcus* (∼9%). It takes part in gluconeogenesis and converts pyruvate back into phosphoenolpyruvate (Berman and Chon, 1970). Although the genera *Phasolarctobacterium* and *Acidaminococcus* are important in the pyruvate to phosphoenolpyruvate conversion, they did not fit into the core microbiome because they were not detectable in all rumen samples.

In the rumen, pyruvate is converted anaerobically to SCFA (Stevens and Hume, 1998) (green arrows in **Figure 6**). The first step in this process is the generation of precursor metabolite acetyl-CoA from pyruvate (Charon et al., 1999). According to our transcriptomic data, pyruvate-flavodoxin oxidoreductase (IPR011895) is the key enzyme in this process from *Prevotella* (∼51%), *Clostridium* (∼13%) and *Bacteroides* (∼5%). Another reaction of pyruvate may lead to the simultaneous formation of formate and acetyl-CoA. This enzyme may participate in protecting the bacteria against oxidative stress (Nakayama et al., 2013). Pyruvate formate lyase (PFL) (IPR012838) catalyzes the conversion of pyruvate and CoA to formate and acetyl-CoA has been found in representatives of *Prevotella* (∼13%), *Veillonellaceae* (∼12%), and *Lachnospiraceae* (∼5%). Formate acetyltransferase (IPR005949) (black arrows in **Figure 6**) acts in the reverse direction, and was detected in the genera *Prevotella* (∼9%), *Butyrivibrio* (∼8%), *Eubacterium* (∼6%), *Succinatimonas* (∼6%), *Treponema* (∼6%) and *Clostridium* (∼4%).

Formate in the rumen serves as a precursor metabolite for hydrogenotrophic methanogenesis (black lines in **Figure 6**) or reductive acetogenesis (Ragsdale and Pierce, 2009; Ungerfeld, 2014). In reductive acetogenesis, the formate-tetrahydrofolate ligase (FTHFS) gene was identified among the core metabolic functions (blue line in **Figure 6**). FTHFS (IPR000559) catalyzes the ATP-dependent activation of formate ions via its addition to the N10 position of tetrahydrofolate (Ragsdale and Pierce, 2009). FTHFS is widely considered indicative of the Wood-Ljungdahl pathway of autotrophic CO_2_ fixation (acetogenesis) and was used to identify homoacetogenic bacteria (Xu et al., 2009; Henderson et al., 2010; Müller et al., 2016) (**Supplementary Figure S4**). Interestingly, the gene encoding this enzyme was largely supplied by an uncultured bacterium (∼75%), followed by *Bifidobacterium* (∼4%), *Bacteroides* (∼3%) and *Ruminococcus* (∼3%). The 75% “uncultured” suggests that there are still substantial unexplored fields of homoacetogenesis (Campanaro et al., 2016; Müller et al., 2016).

Although the genera *Bifidobacterium* and *Bacteroides* contained the FTHFS gene, the enzyme does not exclusively serve the Wood-Ljungdahl pathway in these microbes; it functions rather as a methyltransferase in purine and glycine degradation and in the metabolism of some sulfate-reducing bacteria (Xu et al., 2009; Henderson et al., 2010). The genera *Ruminococcus, Clostridium* (∼1%), *Blautia* (∼1%) and order *Clostridiales* (∼0.5%) include known homoacetogens but they are represented in low transcript frequency (**Supplementary Table S3**).

Along the pathway of heterotrophic acetate production, acetyl-CoA becomes phosphorylated to acetyl-phosphate (Schuchmann and Müller, 2014). The acetate/propionate kinase enzyme (IPR004372) can carry out this reaction. Acetate kinase catalyzes the acetyl-phosphate to acetate reaction with concomitant ATP generation (Hasona et al., 2004). The corresponding transcript was widespread in the genus *Prevotella* (∼69%) followed by *Bacteroides* (∼7%), *Clostridium* (∼3%), *Ruminococcus* (∼3%) and *Lactobacillus* (∼3%). Acetate kinase can be involved both in the reductive acetogenesis and heterotrophic acetate production (Schuchmann and Müller, 2014); thus, the genera attached to this reaction are probably mixed. Butyrate is produced by butyrate kinase (IPR011245) from butyryl-CoA (Louis et al., 2004). The corresponding members of the rumen fluid microbial community were *Prevotella* (∼52%) and *Bacteroides* (∼14%), respectively. Our data indicated that several gene products contributed to the regulation of acetate and butyrate production, i.e., the alpha subunit of acetyl-CoA carboxylase (IPR001095), the beta subunit of acetyl-CoA carboxylase carboxyl transferase (IPR000438) and the acetyl-CoA biotin carboxyl carrier (IPR001249) (green lines in **Figure 6**). These enzymes carry out the carboxylation of acetyl-CoA to malonyl-CoA, thus they tightly regulate the SCFA synthesis (Rock and Jackowski, 2002). The taxa linked to this regulation were the genera *Prevotella* and *Ruminococcus* (**Supplementary Table S3**).

Two gene products were identified in the core functions in the pathway of oxaloacetate biosynthesis. One of them was the phosphoenolpyruvate carboxykinase (IPR001272), which catalyzes the reversible carboxylation of phosphoenolpyruvate to oxaloacetate (purple arrow in **Figure 6**). This has been envisioned as a major carboxylation enzyme for the generation of oxaloacetate intermediate in succinate-producing saccharolytic anaerobes (Samuelov et al., 1991; Schöcke and Weimer, 1997). Our results revealed that the genera *Clostridium* (∼23%), *Ruminococcus* (∼23%), *Treponema* (∼23%) and *Eubacterium* (∼6%) contributed to this particular metabolic pathway. An alternative oxaloacetate synthetic pathway from pyruvate also exists. The pyruvate carboxylase (IPR005930) enzyme is essential in this function, shifting the metabolism toward gluconeogenesis (Jitrapakdee et al., 2008). The reaction involves the irreversible ATP-dependent carboxylation of pyruvate to oxaloacetate; we detected the likely presence of this reaction in the genera *Eubacterium* (∼17%), *Clostridium* (∼17%), *Oscillibacter* (∼17%), and *Megasphaera* (∼11%), respectively.

Further on in this metabolic route, malate is produced from oxaloacetate by malate L-lactate dehydrogenase (IPR003767). This is more favorable than the reverse reaction in bacterial species (Honka et al., 1990) and is carried out by a diverse community of microbial genera, including *Slackia* (∼6%), *Clostridium* (∼5%), *Olsensella* (∼5%), *Blautia* (∼3%), *Escherichia* (∼3%), and *Lactobacillus* (∼3%). Malate and fumarate are precursor metabolites for succinate (purple arrows in **Figure 6**). Succinate plays very important role in the rumen fermentation (Bryant et al., 1957; Wen et al., 1996; Matsui et al., 2000; Russell and Rychlik, 2008; Purushe et al., 2010; Reichardt et al., 2014; Bozan et al., 2017; Deusch et al., 2017). Our results indicated that the core succinate producers in the rumen were representatives of *Clostridium, Eubacterium*, and *Ruminococcus* (Russell and Rychlik, 2008; Reichardt et al., 2014). The ruminal decarboxylation of succinate is rapid and the resulting propionate plays a key role in maintaining glucose homoeostasis (Purushe et al., 2010). Propionate is synthesized via the succinyl-CoA, methylmalonyl-CoA, and propionyl-CoA metabolic pathway (purple arrows in **Figure 6** and **Supplementary Figure S5**). The beta chain of methylmalonyl-CoA mutase (IPR004608) (Takahashi-Iñiguez et al., 2012) was identified, indicating that this is part of the core biochemical pathways in the rumen. This enzyme catalyzes the isomerization of succinyl-CoA to methylmalonyl-CoA and the enzyme activity was carried by the genera *Prevotella* (∼68%), *Phocaeicola* (∼10%) and *Bacteroides* (∼9%). Succinate-CoA transferase (IPR017821) may catalyze the pathway for the decarboxylation of succinate to propionate^5^. The gene coding for this enzyme could be linked to the genera *Prevotella* (∼61%), *Phasolarctobacterium* (∼13%), and *Bacteroides* (∼5%), respectively. The propionate-producing genera *Phocaeicola* and *Phasolarctobacterium* (Al Masalma et al., 2009; Maspolim et al., 2015) did not make it into the core rumen microbiome because some of the rumen samples did not contain these genera in detectable amounts. This implies that the genera *Prevotella* and *Bacteroides* predominate in the metabolic conversion of succinate to propionate.

The principal route to methanogenesis is the hydrogenotrophic one, in which CO_2_, formate and H_2_ are used as substrates (Hook et al., 2010; Janssen, 2010; Morgavi et al., 2012; Wirth et al., 2012; Ungerfeld, 2015; Wallace et al., 2015, 2017; Kamke et al., 2016) (black arrows in **Figure 6**). Reducing equivalents and CO_2_ are released along the fermentation process (Stevens and Hume, 1998; Russell and Rychlik, 2008). Molecular H_2_ is produced actively by hydrogenases. We located in the core functions the [FeFe] hydrogenase maturation HydE gene (IPR024021), which is essential for the maturation the [FeFe] hydrogenase (King et al., 2006). The genera *Roseburia* (∼16%), *Clostridium* (∼14%), *Ruminococcus* (∼9%), *Bacteroides* (∼8%) and *Megasphaera* (∼6%) displayed the corresponding transcripts. Formate, may be used for methanogenesis directly or after splitting it to H_2_ and CO_2_ by the formate hydrogenlyase complex (Russell and Rychlik, 2008; McDowall et al., 2014; Ungerfeld, 2015). H_2_ plays an important role in the overall rumen function and in animal nutrition. Efficient H_2_ removal leads to a nutritionally more favorable assortment of SCFA and to an increased rate of fermentation by eliminating the inhibitory effect of H_2_ on acetogens (Henderson et al., 2010; Petri et al., 2013; Shi et al., 2014; Mao et al., 2015; Ungerfeld, 2015). Our results further corroborated the dominance of hydrogenotrophic methanogenesis in the rumen. In addition, we identified four essential gene transcripts among the core functions, which were related to methanogenesis, i.e., the alpha, beta and gamma subunits of methyl coenzyme M reductase (IPR016212, IPR003179, IPR003178) and 5,10-methylenetetrahydromethanoprotein reductase (IPR019946). All known methanogens express the enzyme methyl coenzyme M reductase, which catalyzes heterodisulfide formation between methyl-coenzyme M and coenzyme B and the subsequent release of methane (Ferry, 1999; Hallam et al., 2003). In our data set, the hydrogenotrophic genus *Methanobrevibacter* expressed these genes, although we also noted an unknown methanogenic archaeon associated with relatively high expression level (**Supplementary Table S3**). This suggested that the ruminal archaeal community has not been fully explored to date (Campanaro et al., 2016). Also, 5,10-methylenetetrahydromethanopterin reductase, an F420-dependent enzyme of methanogenesis (Brömmelstroet et al., 1990, 1991), was linked in our transcriptomic data to *Methanobrevibacter* (∼92%) and *Methanobacterium* (∼6%).

## Conclusion

The metagenome and metatranscriptome of the rumen fluid fractions of ten lactating cows showed a fairly consistent picture, which corroborated and extended our knowledge from similar previous studies. Ten separate rumen samples were analyzed, about one third of the average 305,844 Ion Torrent DNA reads could be associated with taxonomic information in the NCBI Taxonomy database. In the RNAseq experiments an average of 680,886 reads were obtained and approximately half of them predicted proteins of known functions. Each set of data was subjected to quality control and filtering before co-occurrence analysis, which was based on the assumption that those microbes which occurred together were likely to form physiological relationships.

A core microbiome, i.e., a collection of taxa present in all ten sequenced samples, was established. The taxonomic core microbiome consisted of 48 genera belonging in *Bacteria* and 1 *Archaeon*. Based on their relative abundances, about a dozen genera formed the majority of the microbiota; the genus *Prevotella* exceedingly predominated the community. *Fungi* were not placed in the core microbiome, which might suggest that either fungi formed a diverse community or they were present at a very low number in our samples and their DNA sequence reads could not reach the threshold.

Thousands of mRNA sequences pointed to active proteins, implicating a complex and diverse metabolism taking place in the rumen. From the 587 functions, a core functional group was distinguished based on co-occurrence analysis, but only 62 of them could be assigned to metabolic functions. This is clearly a low number to cover all possible metabolic pathways and is ascribed to very stringent filtering conditions and to the selection of common elements in 10 separate rumen fluid samples. Nevertheless, even this minimal functional core revealed key enzymes participating in various metabolic processes. As expected, a diverse and rich collection of enzymes was involved in carbohydrate metabolic processes, but other functional groups were also sufficiently represented. Transcripts coding for enzymes active in methanogenesis made up 1% of the core functions.

The genera associated with the core metabolic functions were identified. The main conclusion drawn from this investigation was that for all metabolic functions, performed primarily by *Bacteria*, several genera could provide the necessary activity. In other words, there always seems to be a “back-up” microbial team to substitute the predominant bacteria to accomplish any given metabolic function. Nevertheless, the key actors in most metabolic functions belong to the genus *Prevotella*. The *Prevotella* predominance is partly due to their massive abundance and partly to the metabolic versatility of this taxon. Contrary to the extensive potential to share the contribution to the tasks among bacterial taxa, methanogenesis seemed to have limited possibilities, i.e., only the hydrogenotrophic pathway existed with a limited possible role for “unidentified” methanogens. The potential bypass biochemical routes make the diversion of metabolism toward advantageous pathways by managing the rumen community composition a considerable challenge. Nonetheless, methane emission mitigation seems conceivable via targeting the hydrogenotrophic genus *Methanobrevibacter*.

## Author Contributions

KK conceived the study, participated in its design and evaluation. GK and JH collected the rumen samples and took part in the assessment of the data. RW, GM, and BK performed the sequencing experiments and statistically evaluated the metagenomic data sets. KK, RW, ZB, GR, and ÁS composed the manuscript. All the authors agreed in publishing the final version of this paper.

## Conflict of Interest Statement

The authors declare that the research was conducted in the absence of any commercial or financial relationships that could be construed as a potential conflict of interest.

## Footnotes

1 http://www.cazy.org

2 https://www.r-project.org

3 https://www.cazypedia.org

4 https://www.ebi.ac.uk/interpro/

5 https://www.ebi.ac.uk/interpro/entry/IPR017821

## Supplementary Material

FIGURE S1 Rarefaction analysis of the metagenome sequencing of the 10 rumen samples (R1–R10).

FIGURE S2 Co-occurrence analysis of the metagenome (DNA) samples. Enlargement of the picture shows the taxon belonging to the colored dot in the network. Taxa identified in the core of all ten rumen samples were included in the calculation.

FIGURE S3 The carbon fixation pathway in prokaryotes (Kegg metabolic map). The blue enzyme numbers have been identified in the RNA dataset.

FIGURE S4 The propionate metabolism pathway in prokaryotes (Kegg metabolic map). The blue enzyme numbers have been identified in the RNA dataset.

FIGURE S5 The butanoate metabolism pathway in prokaryotes (Kegg metabolic map). The blue enzyme numbers have been identified in the RNA dataset.

FIGURE S6 The methanogenesis pathway in prokaryotes (Kegg metabolic map). The blue enzyme numbers have been identified in the RNA dataset.

FIGURE S7 Co-occurrence analysis of the metatranscriptome (RNA) samples. Enlargement of the picture shows the function belonging to the colored dot in the network. Functions identified in the core of all ten rumen samples were included in the calculation.

TABLE S1 Composition of feed received by the cows involved in this study.

TABLE S2 Detailed sequencing data of the metagenomics and metatranscriptomic samples.

TABLE S3 Compilation of all CAZymes identified in the rumen samples.

TABLE S4 List of all functions identified in the core, i.e., present in all 10 rumen samples.
