# Supplementary figures and images for: The Planktonic Core Microbiome and Core Functions in the Cattle Rumen by Next Generation Sequencing
Source: Front Microbiol. 2018 Sep 24;9:2285. doi: 10.3389/fmicb.2018.02285 (PMC6165872; doi:10.3389/fmicb.2018.02285)

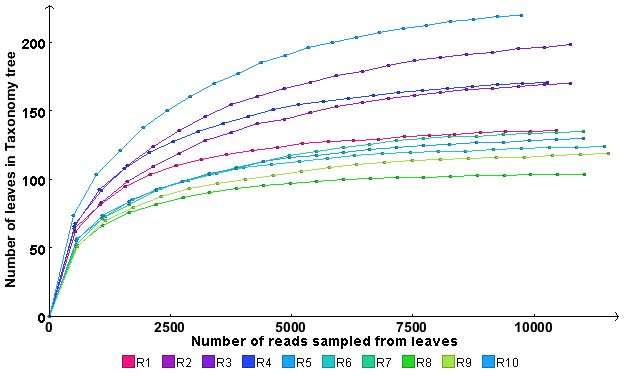

Supplement: FIGURE S1 — Rarefaction analysis of the metagenome sequencing of the 10 rumen samples (R1–R10). [file Image_1.JPEG]

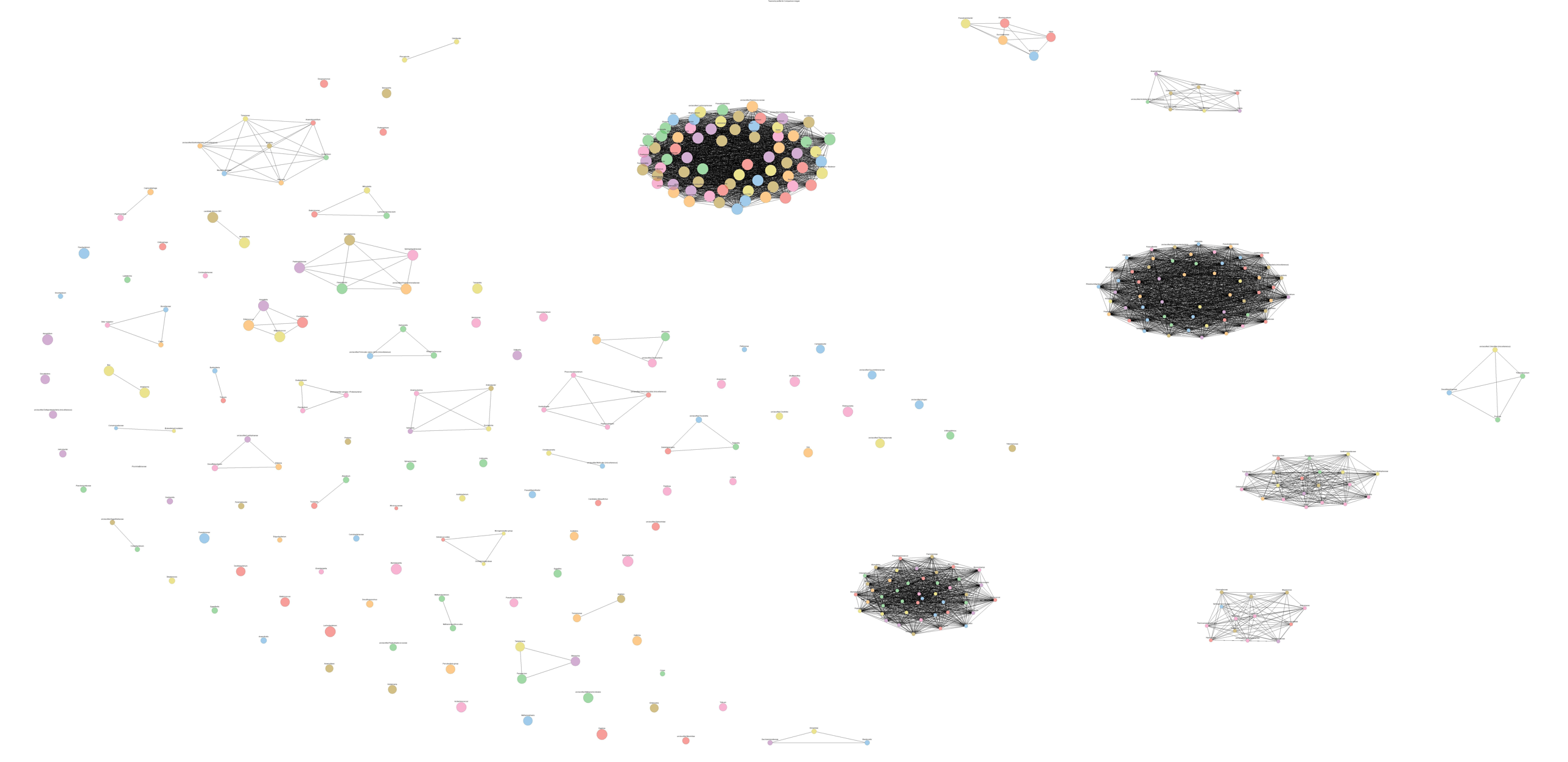

Supplement: FIGURE S2 — Co-occurrence analysis of the metagenome (DNA) samples. Enlargement of the picture shows the taxon belonging to the colored dot in the network. Taxa identified in the core of all ten rumen samples were included in the calculation. [file Image_2.JPEG]

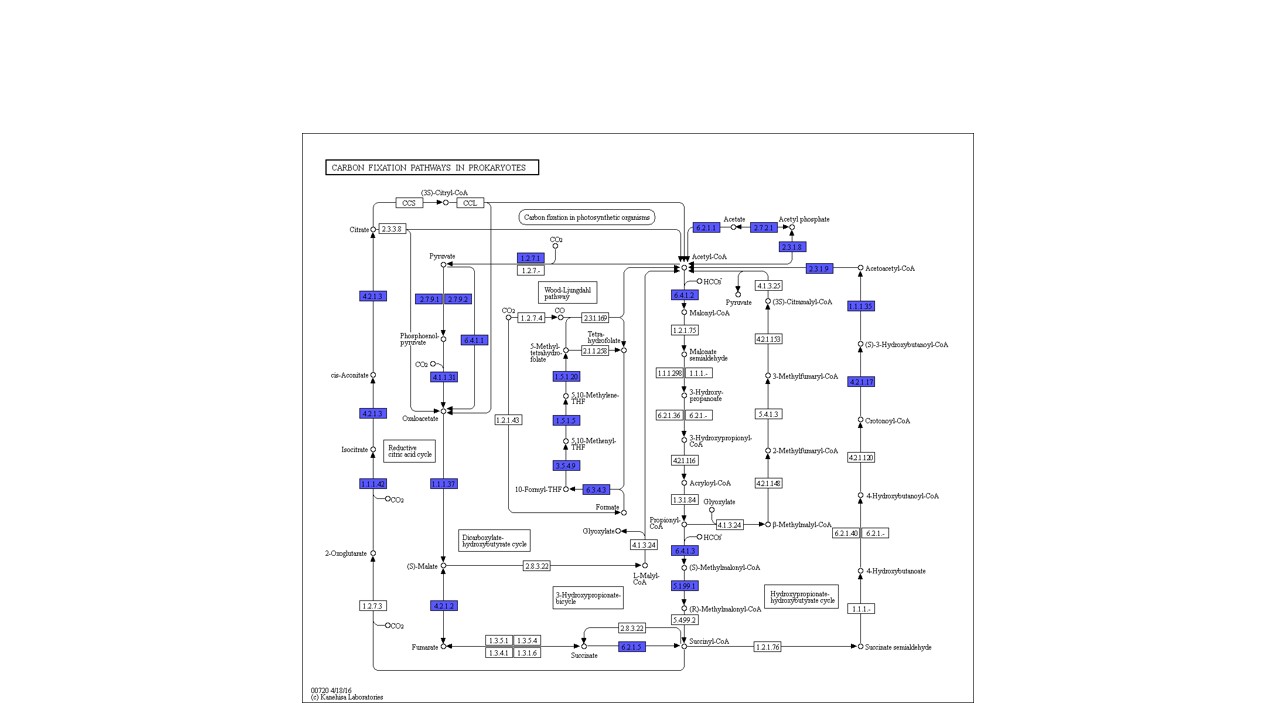

Supplement: FIGURE S3 — The carbon fixation pathway in prokaryotes (Kegg metabolic map). The blue enzyme numbers have been identified in the RNA dataset. [file Image_3.JPEG]

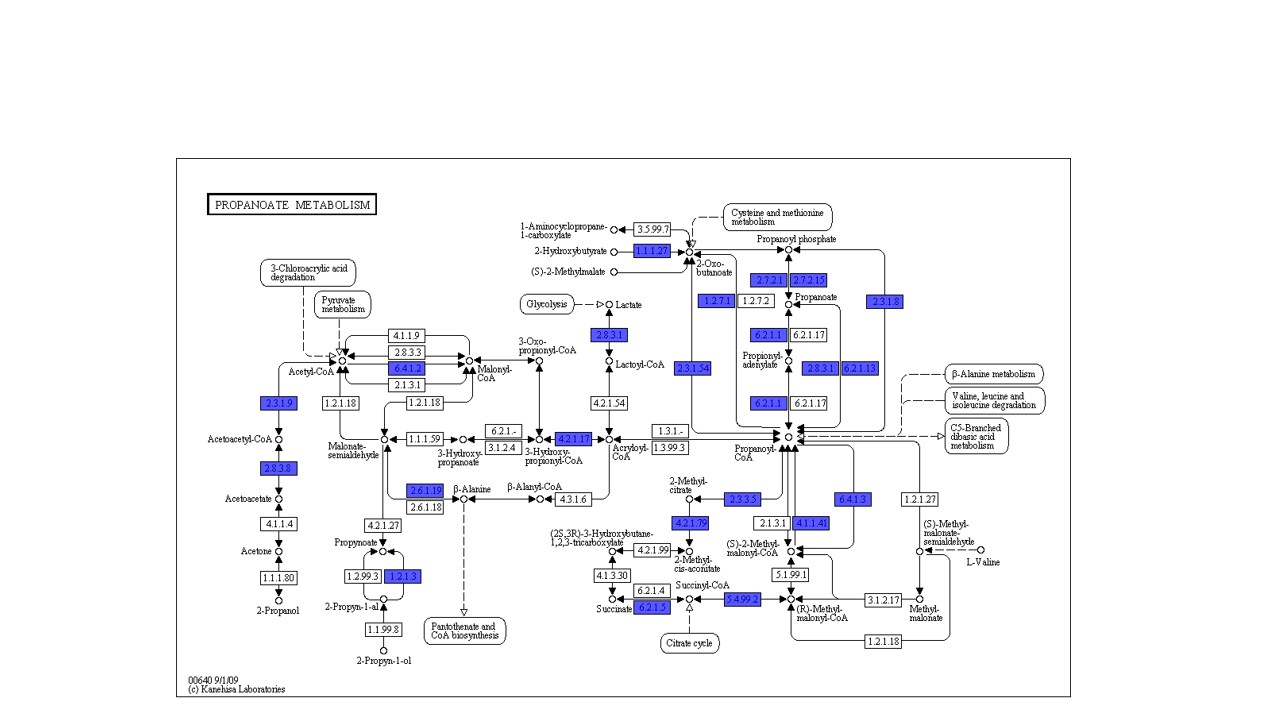

Supplement: FIGURE S4 — The propionate metabolism pathway in prokaryotes (Kegg metabolic map). The blue enzyme numbers have been identified in the RNA dataset. [file Image_4.JPEG]

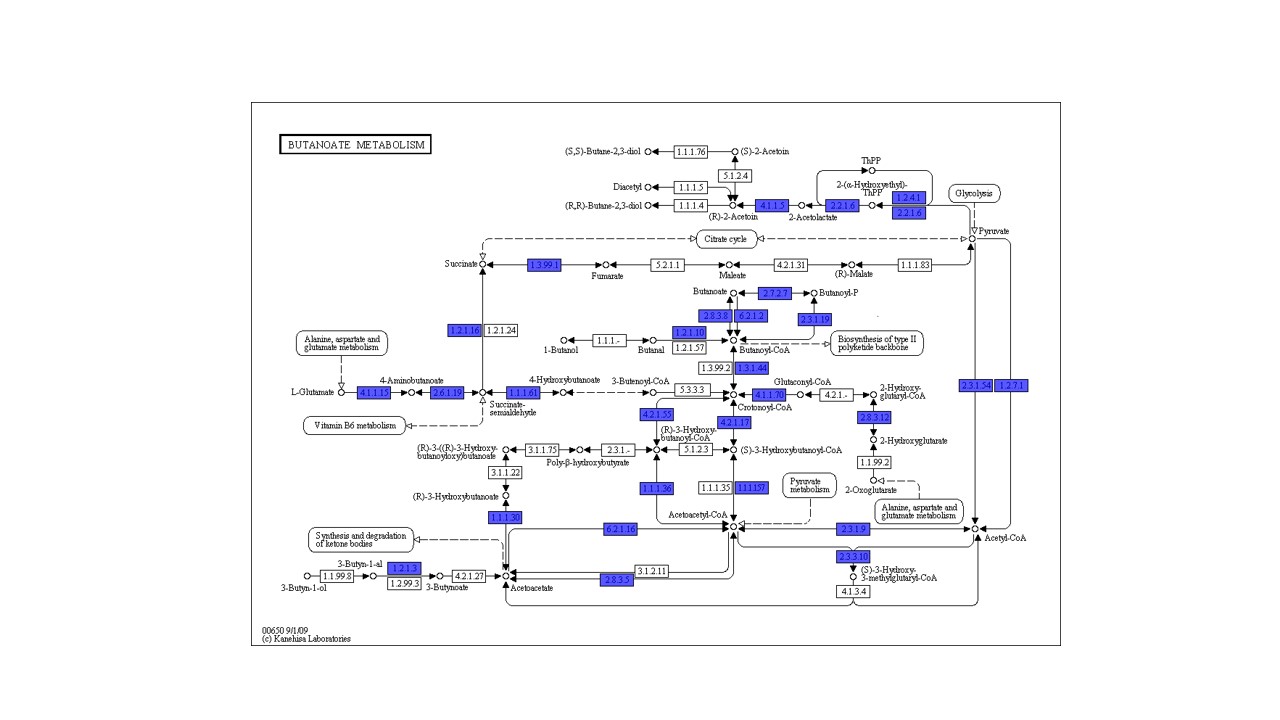

Supplement: FIGURE S5 — The butanoate metabolism pathway in prokaryotes (Kegg metabolic map). The blue enzyme numbers have been identified in the RNA dataset. [file Image_5.JPEG]

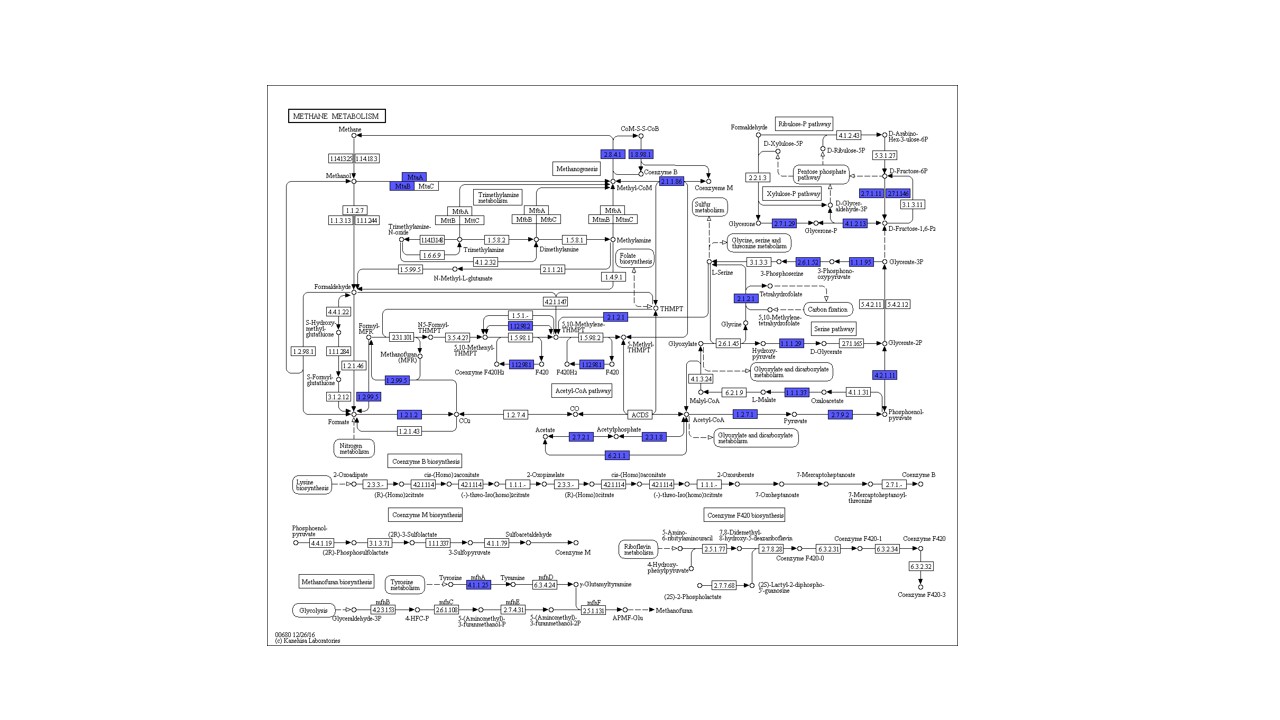

Supplement: FIGURE S6 — The methanogenesis pathway in prokaryotes (Kegg metabolic map). The blue enzyme numbers have been identified in the RNA dataset. [file Image_6.JPEG]

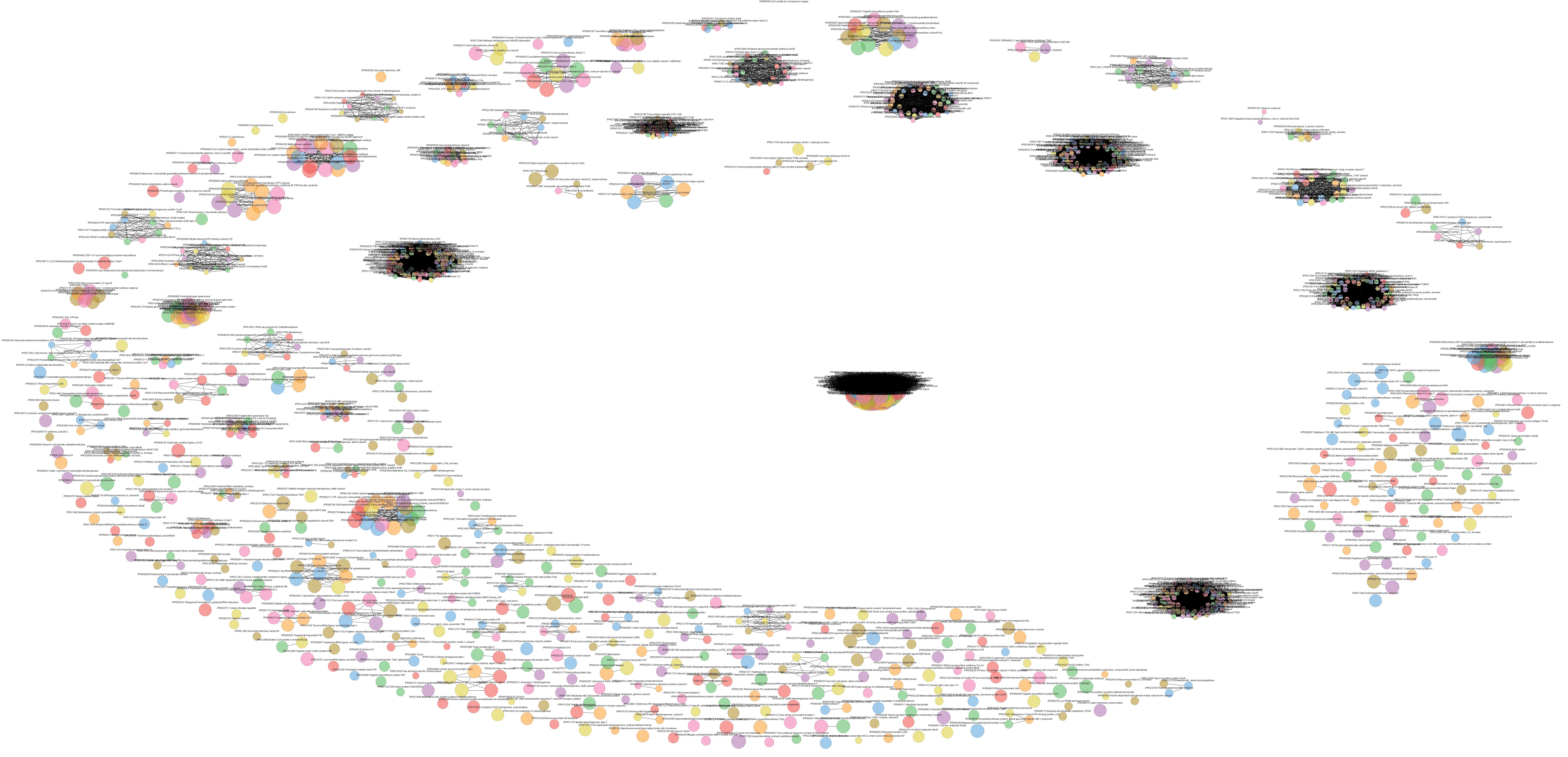

Supplement: FIGURE S7 — Co-occurrence analysis of the metatranscriptome (RNA) samples. Enlargement of the picture shows the function belonging to the colored dot in the network. Functions identified in the core of all ten rumen samples were included in the calculation. [file Image_7.JPEG]
