# Supplementary material for: The Planktonic Core Microbiome and Core Functions in the Cattle Rumen by Next Generation Sequencing
Source: Front Microbiol. 2018 Sep 24;9:2285. doi: 10.3389/fmicb.2018.02285 (PMC6165872; doi:10.3389/fmicb.2018.02285)
Supplement: TABLE S2 — Detailed sequencing data of the metagenomics and metatranscriptomic samples. [file Table_2.DOCX]

| Sample names | Upload: bp count | Post QC: bp count | Post QC: read number | Post QC: read length |
| --- | --- | --- | --- | --- |
| DNA |  |  |  |  |
| Cow 1 | 20,717,420 | 20,642,760 | 116,850 | 177 |
| Cow 2 | 13,063,754 | 13,010,139 | 76,073 | 171 |
| Cow 3 | 16,813,092 | 16,721,633 | 104,833 | 160 |
| Cow 4 | 17,236,316 | 17,172,372 | 97,406 | 176 |
| Cow 5 | 14,438,789 | 14,405,171 | 81,946 | 176 |
| Cow 6 | 54,559,177 | 54,462,447 | 278,569 | 196 |
| Cow 7 | 17,524,965 | 17,461,366 | 102,419 | 170 |
| Cow 8 | 281,628,664 | 280,418,156 | 1,617,918 | 173 |
| Cow 9 | 42,942,040 | 42,772,851 | 245,482 | 174 |
| Cow 10 | 26,019,839 | 25,935,232 | 146,362 | 177 |
| RNA |  |  |  |  |
| Cow 1 | 138,826,763 | 137,543,661 | 769,026 | 179 |
| Cow 2 | 198,508,378 | 197,696,063 | 961,477 | 206 |
| Cow 3 | 156,737,508 | 155,941,201 | 800,102 | 195 |
| Cow 4 | 61,573,345 | 61,239,637 | 348,594 | 176 |
| Cow 5 | 32,789,300 | 32,666,416 | 181,138 | 180 |
| Cow 6 | 174,101,332 | 173,816,296 | 855,351 | 203 |
| Cow 7 | 89,286,304 | 88,404,795 | 526,945 | 168 |
| Cow 8 | 164,036,136 | 162,882,067 | 811,339 | 201 |
| Cow 9 | 141,767,852 | 140,681,121 | 688,496 | 204 |
| Cow 10 | 119,777,298 | 119,576,402 | 670,633 | 178 |
